# Isolation and Characterization of CD34+ Blast-Derived Exosomes in Acute Myeloid Leukemia

**DOI:** 10.1371/journal.pone.0103310

**Published:** 2014-08-05

**Authors:** Chang Sook Hong, Laurent Muller, Michael Boyiadzis, Theresa L. Whiteside

**Affiliations:** 1 University of Pittsburgh Cancer Institute, Pittsburgh, Pennsylvania, United States of America; 2 University Hospital, Department of Otolaryngology and Head & Neck Surgery, Basel, Switzerland; 3 University of Pittsburgh School of Medicine, Division of Hematology-Oncology, Pittsburgh, Pennsylvania, United States of America; 4 University of Pittsburgh School of Medicine, Departments of Pathology, Immunology and Otolaryngology, Pittsburgh, Pennsylvania, United States of America; UT MD Anderson Cancer Center, United States of America

## Abstract

Exosomes are membrane-bound vesicles found in all biological fluids. AML patients' plasma collected at diagnosis contains elevated exosome levels relative to normal donor (ND) plasma. The molecular profile of AML exosomes changes in the course of therapy and may serve as a measure of disease progression or response to therapy. However, plasma contains a mix of exosomes derived from various cell types. To be able to utilize blast-derived exosomes as biomarkers for AML, we have developed an immunoaffinity-based capture method utilizing magnetic microbeads coated with anti-CD34 antibody (Ab). This Ab is specific for CD34, a unique marker of AML blasts. The capture procedure was developed using CD34+ exosomes derived from Kasumi-1 AML cell culture supernatants. The capture capacity of CD34microbeads was shown to linearly correlate with the input exosomes. A 10 uL aliquot of CD34 microbeads was able to capture all of CD34+ exosomes present in 100–1,000 uL of AML plasma. The levels of immunocaptured CD34+ exosomes correlated with the percentages of CD34+ blasts in the AML patients' peripheral blood. The immunocaptured exosomes had a typical cup-shaped morphology by transmission electron microscopy, and their molecular cargo was similar to that of parental blasts. These exosomes were biologically-active. Upon co-incubation with natural killer (NK) cells, captured blast-derived exosomes down-regulated surface NKG2D expression, while non-captured exosomes reduced expression levels of NKp46. Our data provide a proof-of-principle that blast-derived exosomes can be quantitatively recovered from AML patients' plasma, their molecular profile recapitulates that of autologous blasts and they retain the ability to mediate immune suppression. These data suggest that immunocaptured blast-derived exosomes might be useful in diagnosis and/or prognosis of AML in the future.

## Introduction

Although advances in acute myeloid leukemia (AML) therapy have considerably improved initial response rates and patients' quality of life, survival rates remain low, largely due to disease relapse [1], [2]. The failure of conventional therapies to control leukemic relapse has stimulated the search for novel, more effective therapeutic approaches. Recently, tumor-derived exosomes (TEX) have emerged as one of the key modulators of immune evasion/suppression in cancer, including hematologic malignancies [3]–[6]. In view of emerging evidence for the critical role of the host immune system in cancer progression, evaluation of the impact TEX exert on the disease activity, severity and response to therapy in AML is of great interest.

Exosomes are 30 to 100 nm intraluminal vesicles (ILV) generated by inward budding of endosomal multivesicular bodies (MVB). MVB traffic to and fuse with the plasma membrane, releasing ILV into the extracellular space [7]. The exosomal cargo includes proteins/glycoproteins expressed on the cell membrane as well as molecules and soluble factors present in the cytosol of parental cells. Exosomes are found in all biological fluids, including urine, plasma or ascites. While exosome secretion occurs under physiologic conditions, and all cells are capable of their release, tumor cells are avid exosome producers. Importantly, exosome fractions obtained from plasma of cancer patients are enriched in molecules which play a role in tumor proliferation, immune escape and metastasis [8]–[12].

Plasma of newly-diagnosed AML patients contains higher levels of exosomal protein (in µg/mL plasma) than normal donors' (ND) plasma [13], [14]. Also, AML exosomes have a distinct molecular profile. In addition to conventional exosome markers, such as tetraspanins, they contain membrane-associated TGF-β1, MICA/MICB, FasL and myeloid blast markers, CD33, CD34 and CD117 [13]. When tested *ex vivo*, AML exosomes decreased natural killer (NK)-cell cytotoxicity by down-regulating NKG2D expression levels in normal NK cells [13], [14]. Antibody neutralization of TGF-β1 carried by AML exosomes partially restored NK-cell functions, suggesting that exosome-associated TGF-β1 is in part responsible for NK-cell dysfunction in AML. Thus, exosomes present in AML patients' plasma have a potential to modulate innate immune responses and could influence disease progression or response to therapy.

Exosomes are purified from biological fluids using differential centrifugation, ultrafiltration, size-exclusion chromatography and high-speed ultracentrifugation [13], [14]. However, exosomes isolated from human plasma are largely derived from normal cells, such as e.g., platelets, which are known to be strong exosome producers [15]. TEX, if present in plasma, probably account for only a fraction of all isolated exosomes. We hypothesize that AML blast-derived exosomes are more biologically active than exosomes produced by normal cells and that they largely mediate immune suppression. To be able to perform molecular analyses of blast-derived exosomes, establish their clinical significance and determine their role as potential biomarkers of disease progression/relapse, it is first necessary to separate them from plasma exosomes derived from normal cells. Because AML blast-derived exosomes carry a unique set of membrane-associated molecules, which mimic those present in the membrane of AML blasts, they can be distinguished and separated from non-blast exosomes present in the same plasma. Based on this principle, we have developed an immunoaffinity-based capture method for AML blast-derived exosomes using microbeads coated with anti-CD34 antibody (Ab). Our data provide a proof-of- principle that blast-derived exosomes can be quantitatively recovered from AML patients' plasma and that their molecular profile recapitulates that of the blasts. These isolated exosomes are biologically-active, mediate immune suppression and might be useful in AML diagnosis and/or prognosis in the future.

## Materials and Methods

### Cell culture supernatants

A human CD34+ leukemic cell line (Kasumi-1) was purchased from American Type Culture Collection (Manassas, VA, USA) and cultured in RPMI supplemented with 2 mM/ml L-glutamine, 100 U/ml penicillin, 100 ug/ml streptomycin and 10% (v/v) fetal bovine serum (FBS). All reagents were purchased from Invitrogen (Grand Island, NY, USA). FBS was depleted of bovine exosomes by ultracentrifugation at 100,000×g for 3 hrs. Conditioned media (CM) were collected from 48–72 hr sub-confluent cell cultures after centrifugation at 10,000 g for 30 min and concentrated using a Vivacell 100 unit (Sartorius Corp, Bohemia, NY, USA). Concentrated CM was filtered using a 0.22 um syringe-filter unit and used for exosome isolation.

### Plasma of AML patients and healthy donors

Samples of venous blood (20–50 mL) were obtained from newly-diagnosed AML patients prior to any treatment and from age-matched healthy volunteers. All subjects participating in this study signed an informed consent approved by the Institutional Review Board of the University of Pittsburgh (IRB #960279, IRB #0403105 and IRB #0506140). Peripheral blood was collected into heparinized vacutainer tubes, and the samples were hand-carried to the laboratory. Plasma was centrifuged at 1000×g for 10 min to remove blood cells and cell fragments. Clear plasma was collected and immediately used for exosome capture or aliquoted into vials, which were stored in liquid N_2_. Frozen and thawed plasma was centrifuged at 10,000 g for 30 min and used for exosome isolation and for immunoaffinity capture as described below.

### Exosome isolation from plasma or cell supernatants

Exosomes were isolated from CM of cultured cells or from clarified plasma of ND or AML patients as previously described [13], [14] with minor modifications. Briefly, following centrifugation at 10,000×g for 30 min, aliquots of plasma (up to 9 mL) were passed through a 0.22 µm pore size filter and applied to Bio-Gel A50 m columns (Bio-Rad Laboratories, Hercules, CA, USA) packed with Sepharose 2B (Sigma-Aldrich, St. Louis, MO, USA). The column was eluted with phosphate buffered saline (PBS). Fractions between 10 and 28 mL (the void volume peak), which contained proteins of more than 50 million kDa, were collected into a Beckman Optiseal Centrifuge Tube and centrifuged in a Beckman Optima LE-80K Ultracentrifuge (Beckman Coulter, Atlanta, GA, USA) at 100,000×g for 2 h at 4°C. The pellet was resuspended in PBS (200 µL) and analyzed using a protein assay kit (Bio-Rad Laboratories, Hercules, CA, USA).

### Immunoaffinity capture of exosomes from plasma

To isolate blast-derived exosomes from plasma of patients with CD34^+^ blasts, the CD34 microbead kit (Miltenyi Biotec, Auburn, CA, USA) was used. From 0.1 to 1 mL of freshly-thawed plasma was used for immune capture. Exosomes captured on CD34 Ab-coated magnetic beads were recovered from the magnetic column by eluting with Tris Buffered Saline (TBS) as described by the manufacturer (MS column, Miltenyi Biotec, Auburn, CA, USA). Exosome-coated beads were pelleted by centrifugation at 10,000×g for 30 min. Captured exosomes were visualized by transmission electron microscopy (TEM), co-cultured with NK cells or lysed in 2x Laemmli sample buffer for Western blot analyses. In separate experiments, CD61^+^ exosomes were also captured from patients' plasma using CD61 microbead kits (Miltenyi Biotec, Auburn, CA, USA) to capture and remove platelet-derived exosomes.

### Transmission electron microscopy

TEM was performed at the Center for Biologic Imaging at the University of Pittsburgh. Freshly-isolated exosomes were put on a copper grid coated with 0.125% Formvar in chloroform. The grids were stained with 1% v/v uranyl acetate in ddH2O and the samples were examined immediately. A JEOL 1011 transmission electron microscope was used for imaging.

### Western blots

Isolated or immunocaptured exosomes were tested for the presence of CD63, CD81, CD34, GAPDH, CD200, CD44 and CD105 using western blots as previously described [14]. Briefly, 10 µg of exosomes were lysed with Laemmli sample buffer (Bio-Rad Laboratories, Hercules, CA, USA), separated on 7–15% SDS/PAGE gels and transferred onto PVDF membrane (Millipore, Billerica, MA, USA) for western blot analysis. Membranes were incubated overnight at 4°C with antibodies specific for: CD63 (1∶200, sc-15363, Santa Cruz, CA, USA), CD34 (1∶2000, ab81289, Abcam, Cambridge, MA, USA), CD81 (1∶200, PA5-13582, Thermo Fisher, Pittsburgh, PA, USA), GAPDH (1∶500, FL-335, Santa Cruz, CA, USA), CD200 (1∶2000, AF2724, R&D, Minneapolis, MN, USA), CD44 (1∶1000, ab41478, Abcam, Cambridge, MA, USA), CD105 (1∶1000, ab169545, Abcam, Cambridge, MA, USA), platelet IIb/IIIa (1∶200, sc-73544, Santa Cruz, CA, USA). Next, the HRP-conjugated secondary antibody (1∶5000, Pierce, Thermo Fisher, Pittsburgh, PA, USA) was added for 1 hr at room temperature (RT) and blots were developed with ECL detection reagents (GE Healthcare Biosciences, Pittsburgh, PA, USA). The intensities of the bands on exposed films were quantified using Image J software (NIH, USA).

### Flow cytometry

Kasumi-1 cells (2×10^5^) were labeled with phycoerythrin (PE)-conjugated anti-CD33, CD34, CD117 or CD81 antibodies (all from Beckman Coulter, Atlanta, GA, USA) for 20 minutes at 4°C and analyzed using a 4-color Beckman Coulter XL, and data were analyzed using the Expo32-Software.

### Functional studies with captured CD34+ exosomes

Normal human NK cells were co-incubated with isolated exosomes to determine expression levels of the natural cytotoxicity receptor NKp46 and of NKG2D on the surface of these cells as previously described [13.14]. Briefly, NK cells (1×10^5^) were co-incubated for 24 h with CD34+ exosomes captured directly from 1 mL AML plasma or unbound CD34^negative^ exosomes. Then, flow cytometry for expression of NKG2D or NKp46 was performed and data expressed as mean fluorescence intensity (MFI). NK cells incubated in medium without exosomes were used as controls. Isolation of human NK cells from normal donor buffy coats using AutoMACS (Miltenyi Biotec, Auburn, CA, USA) was previously described [13], [14]. PE-conjugated anti-NKG2D and anti-NKp46 antibodies or isotype controls were purchased from Beckman Coulter (Atlanta, GA, USA).

## Results

### Immunoaffinity exosome capture

**Figure 1:**
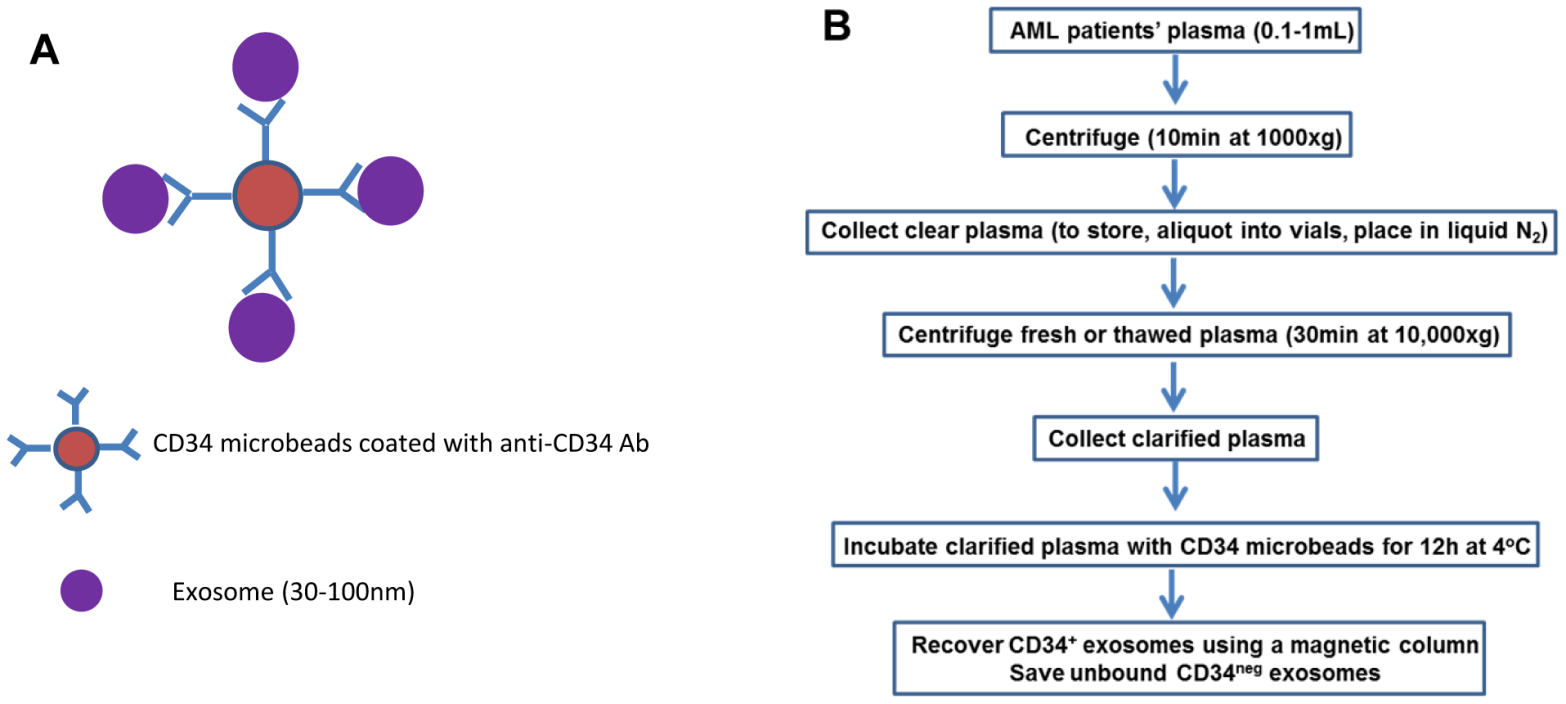
The strategy for capture of CD34^+^ exosomes using anti-CD34 Ab-coated microbeads purchased from Miltenyi (A) and a schema for capture of CD34^+^ exosomes directly from AML patients' plasma (B).

The strategy used for exosome capture from patients' plasma is illustrated in Figure 1A. Freshly harvested or frozen/thawed plasma was processed as shown in Figure 1B, yielding the captured CD34+exosome fraction and non-captured CD34neg exosomes.

### Characteristics of Kasumi-1 cell line-derived exosomes

**Figure 2:**
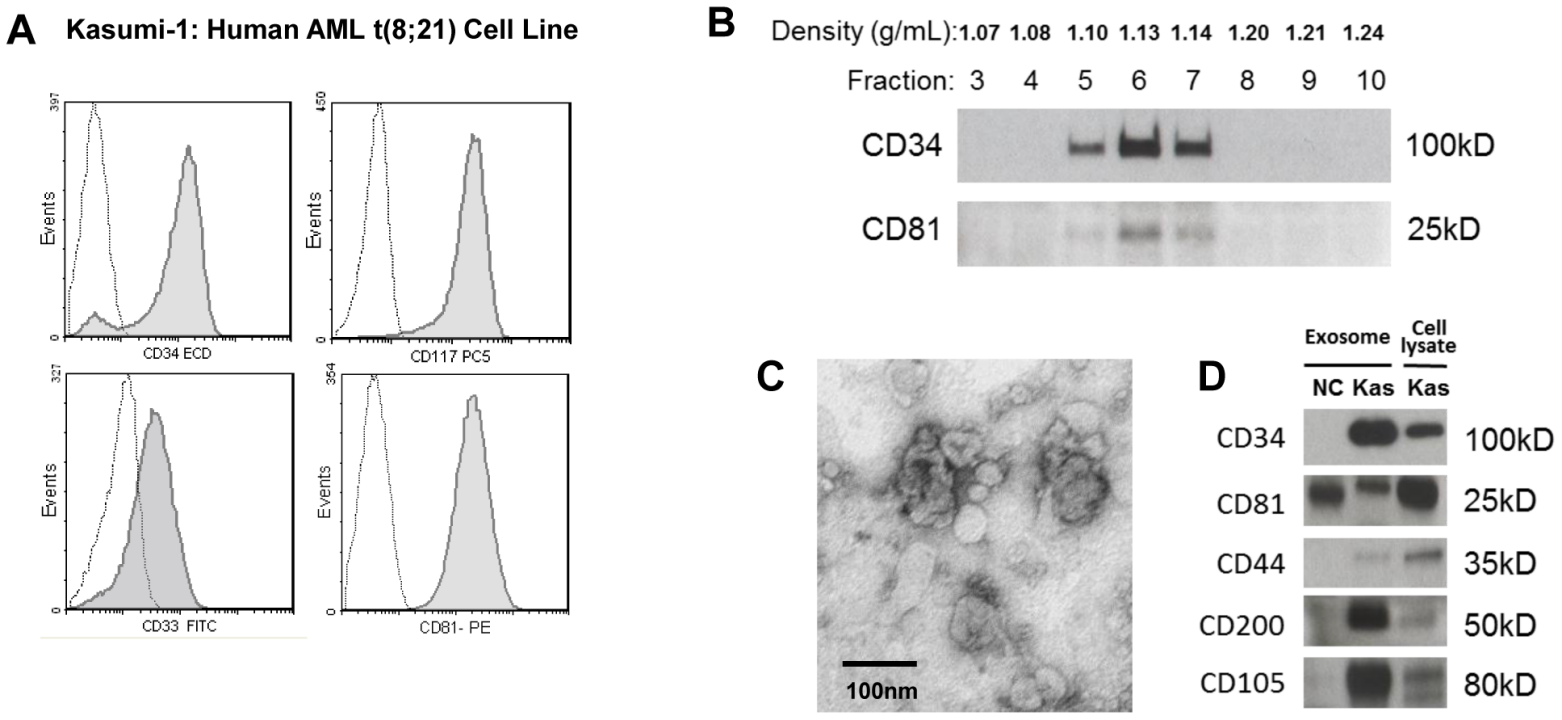
Studies with the Kasumi-1 cell line and with exosomes derived from Kasumi-1 culture supernatants. A) Flow cytometry of Kasumi-1 cells shows expression of blast markers, CD34, CD117 and CD33 and of CD81 (tetraspanin). Dotted lines indicate isotype controls. B) Kasumi-1 exosomes were separated on a continuous sucrose density gradient, and the collected gradient fractions were tested in Western blots for CD34 and CD81. CD34^+^ and CD81^+^ exosomes were recovered at the density of 1.10–1.14. C) An electron microscope image of isolated and negatively stained Kasumi-1 exosomes. D) Western blots of isolated Kasumi-1 exosomes (Kas) and of exosomes isolated from plasma of a normal donor (NC). Each lane was loaded with 10 µg exosomal protein.

Kasumi-1 cell line is a leukemic cell line established from peripheral blood of an AML patient with 8; 21 chromosome translocation. It originated from early myeloid stem cell [16], [17] and expresses leukemic blast markers, including CD34, CD33, CD117 (Figure 2A). Exosomes present in supernatants of cultured Kasumi-1 cells were initially isolated by ultracentrifugation and used to implement the strategy shown in Figure 1A. Isolated Kasumi-1 exosomes floated at density of 1.10–1.14 on a continuous sucrose density gradient (Figure 2B) and were visualized by TEM as typical cup-shaped vesicles measuring 30–70 nM in diameter (Figure 2C). In Western blots, their protein profile included CD34, a blast marker, as well as CD81, the tetraspanin used as an exosomal marker (Figure 2D). Exosomes isolated from supernatants of Kasumi cells recapitulated the molecular profile of the Kasumi cell line (Figure 2D). In addition, Kasumi-1 exosomes also carried proteins known to be expressed in leukemia stem cells: CD44, CD200 and CD105 (Figure 2D). Exosomes isolated from ND plasma were not CD34^+^ (Figure 2D).

### Development of immunocapture using CD34^+^ exosomes isolated from Kasumi-1 supernatants

**Figure 3:**
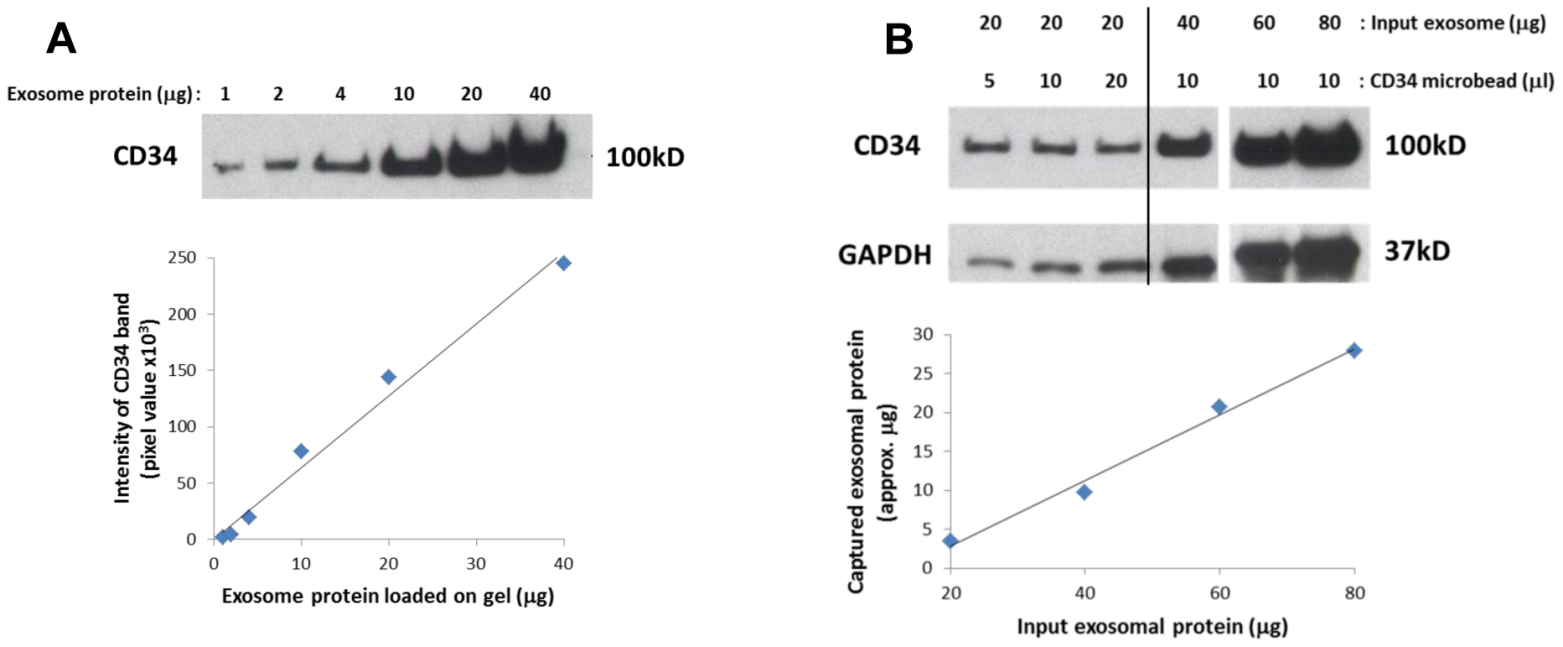
Calibration experiments with CD34^+^ Kasumi-1 exosomes isolated by ultracentrifugation. A) Isolated exosomes were loaded at increasing protein concentrations onto gels and blotted using anti-CD34 Ab. Intensity of each band was measured in pixels. The graph illustrates a linear relationship between exosomal protein levels and pixel intensity in Western blots. B) Isolated exosomes (20 µg protein) were added to increasing volumes of CD34 microbeads *(left*). Five or 10 µL of microbeads were sufficient to capture 20 µg of exosomes. Next, a 10 µL aliquot of beads was used to capture increasing concentrations of Kasumi-1 exosomes (*right*). The graph shows that the capacity of CD34 microbeads to capture up to 80 µg of input exosomes increased linearly. However, at 80 µg only about 40% of exosomal proteins were captured, suggesting that additional microbeads are necessary for capture of all Kasumi-1 exosomes.

**Figure 4:**
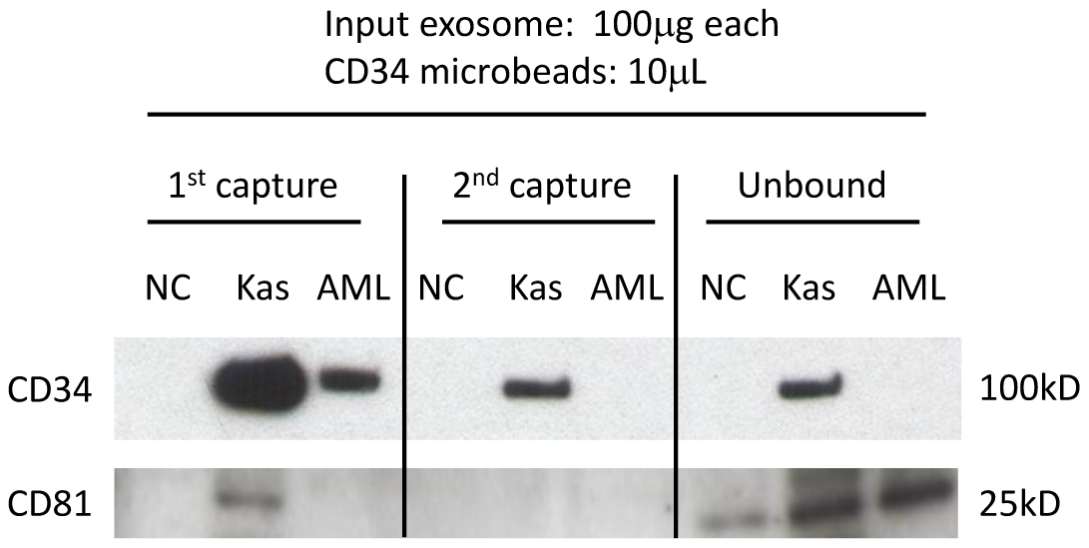
Capture of CD34+ exosomes from total exosomal fractions by CD34+ microbeads. Isolated Kasumi-1 exosomes (Kas) and total exosome fractions isolated by ultracentrifugation from normal donor plasma (NC) or from CD34^+^ AML patient plasma (AML) were used for capture with CD34 microbeads. After the 1^st^ capture and removal of beads, 2^nd^ capture was performed with a fresh aliquot of CD34 microbeads. The final unbound fractions were ultracentrifuged to collect remaining exosomes. While the 2^nd^ capture was necessary to recover all CD34^+^ Kasumi-1 exosomes, a single capture was sufficient to recover all CD34^+^ exosomes from the bulk of exosomes isolated from the AML plasma. There were no CD34^+^ exosomes captured from the bulk exosomal fraction isolated from a normal donor's plasma. CD81 expression indicates that the unbound fractions still contain CD34^neg^ exosomes.

Anti-CD34 Ab-coated microbeads were used to establish optimal conditions for capture of CD34+ exosomes from supernatants of Kasumi-1 cells. First, exosomes were isolated from Kasumi-1 supernatants by ultracentrifugation. The capture capacity of microbeads was calibrated using densitometric analysis of western blot bands reactive with anti-CD34 Ab. As shown in Figure 3A, pixel intensity of each CD34 band linearly correlated with the exosomal protein level loaded on the SDS/PAGE gel. A 5 to10 µL aliquot of CD34 Ab-coated microbeads was able to capture all CD34^+^ exosomes from 20 µg of isolated Kasumi-1 exosomes (Figure 3B, *left*). The beads capture capacity was not improved when the bead concentration was increased to 20 µL (Figure 3B, *left*). Increasing the protein levels of the input exosomes linearly correlated with the protein level of captured CD34^+^ exosomes (Figure 3B, *right and graph*). However, capture efficiency was only about 40% (Figure 3B, *graph*). To improve capture efficiency, additional CD34 microbeads were added after the removal of captured CD34^+^ exosomes. As shown in Figure 4, this second step resulted in capture of additional Kasumi-1-derived CD34^+^ exosomes, but the final *unbound* exosomal fraction still contained CD34^+^ exosomes. However, AML plasma-derived CD34^+^exosomes were completely captured by the first co-incubation with microbeads (Figure 4). The unbound fraction did not contain any CD34^+^ exosomes, although it did contain other exosomal proteins such as CD81. Control exosomes derived from ND plasma did not contain CD34^+^ exosomes. While two repetitions of immunocapture with 10 µL of CD34 microbeads each were necessary to capture about 75% CD34^+^ exosomes present in 100 µg of supernatant-derived exosomes, one 10 µL aliquot of CD34 microbeads was sufficient for capture of all CD34^+^ exosomes from 100 µg of exosomes obtained from AML plasma.

### Capture of CD34^+^ blast-derived exosomes directly from AML plasma

**Figure 5:**
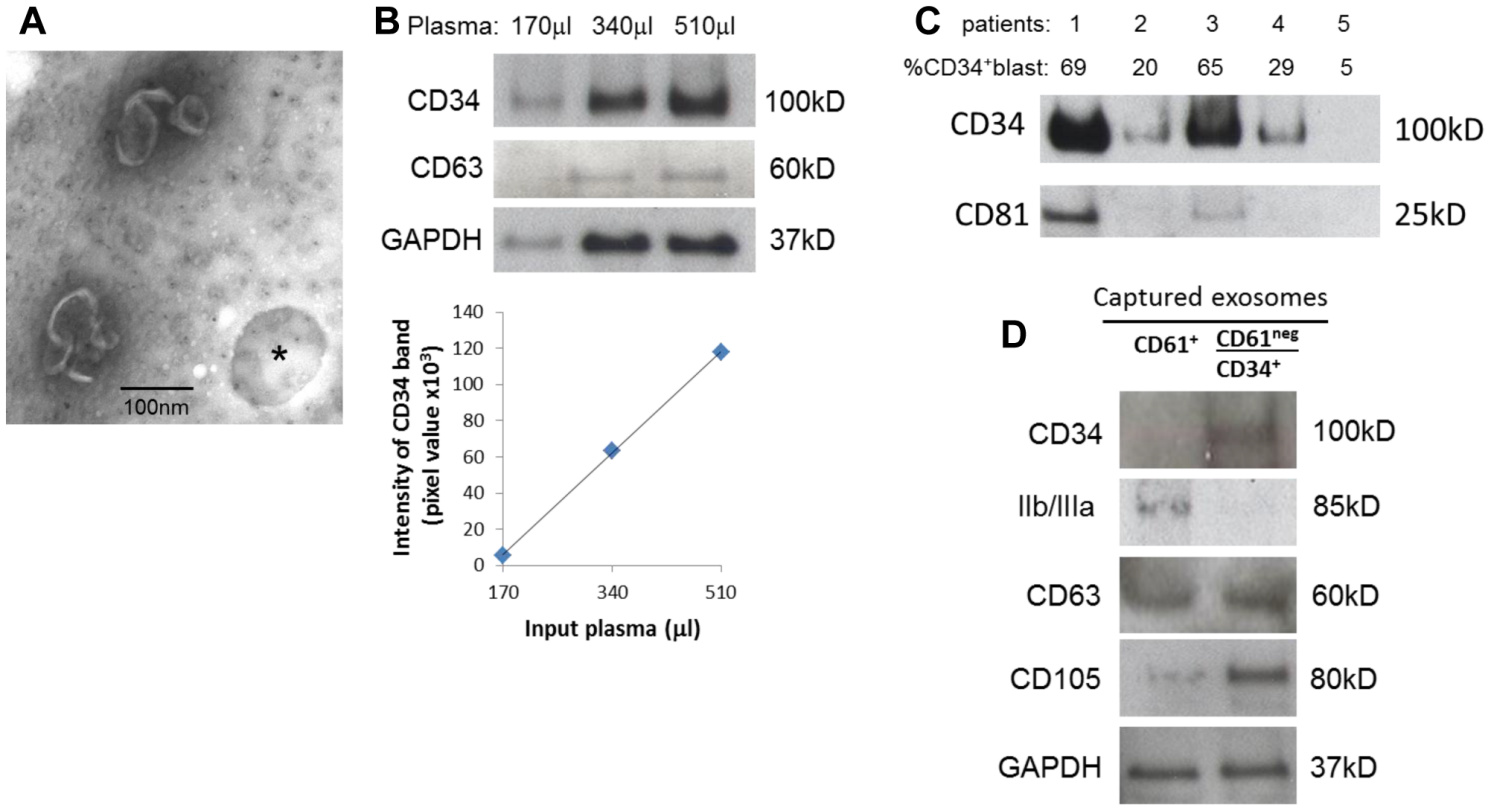
Capture of CD34^+^ blast-derived exosomes directly from AML patients' plasma. A) Negatively stained electron microscope images of exosomes captured on CD34 microbeads (* shows a vacant microbead). B) Increasing AML plasma volumes were used for capture of CD34^+^ exosomes by microbeads and recovered exosomes were studied by Western blots. The graph shows a linear relationship between the input plasma volumes and pixel densities of captured and blotted CD34^+^ exosomes. C) Exosomes were captured from plasma samples obtained from five CD34^+^ AML patients and were analyzed by Western blots. The percentage of leukemic blasts in the peripheral circulation of each of the patients is shown. CD81 serves as the exosome marker. D) Removal of platelet-derived exosomes from plasma using anti-CD61 Ab-coated microbeads prior to capture of CD34^+^ exosomes. Exosomes captured with CD61 microbeads (*left*: CD61^+^) and CD34^+^ exosomes captured after removing CD61^+^ exosomes (*right*: CD61neg/CD34^+^) are shown in a representative Western blot of three evaluated.

Anti-CD34 Ab-coated microbeads were next used to capture exosomes directly from AML patients' plasma. For direct exosome capture, an aliquot (0.1–1 mL) of freshly-harvested or frozen/thawed plasma was co-incubated with a 10 µL aliquot of microbeads coated with anti-CD34 Ab. CD34+ exosomes were captured using a magnetic column as described in Materials and Methods (Figure 1). The TEM images of the recovered fraction showed cup-shaped exosomes attached to microbeads (Figure 5A). The capture capacity of CD34^+^ exosomes from AML plasma correlated linearly with the input volume (in µL) of plasma (Figure 5B). Therefore, using 10 µL of CD34 microbeads, we were able to capture all CD34^+^ exosomes from 100 µL to 1000 µL of AML patients' plasma. This capture procedure was reproduced with five different AML plasma- and five ND plasma- derived exosomes (data not shown). To determine whether protein levels of captured CD34^+^ exosomes reflect the percentage of CD34^+^ blasts in the peripheral circulation of AML patients, exosomes were captured from plasma of 5 patients with high or low blast counts as shown in Figure 5C. Patients with ∼60% circulating CD34^+^ blasts had the highest exosome recovery relative to the patients with fewer blasts in the periphery. Incidentally, CD34+ exosomes carry highly variable quantities of CD81, as shown, e.g., in Figure 5C. For this reason, we prefer to use GAPDH or CD63 as markers of AML exosomes (Figures 5B or D).

### Potential interference of platelet-derived exosomes

Since platelets are one of the major producers of exosomes found in plasma [15], an attempt was made to determine whether platelet-derived exosomes non-specifically bind to CD34 microbeads. We attempted to remove platelet-derived exosomes from plasma using anti-CD61 Ab-coated microbeads. These beads effectively captured exosomes positive for glycoprotein IIb/IIIa as seen in Figure 5D *(left*). Following the removal of CD61^+^ exosomes, CD34 microbeads were added to the remaining plasma to capture CD34+ exosomes. The recovered CD34^+^ exosomes did not contain glycoprotein IIb/IIIa (Figure 5D, right). This experiment showed that unwanted exosomes, which potentially could interfere with capture of CD34+ exosomes or compromise their purity, may be removed by an additional immunocapture step with selected Ab-coated microbeads.

### Characteristics of captured CD34^+^ blast-derived exosomes

**Figure 6:**
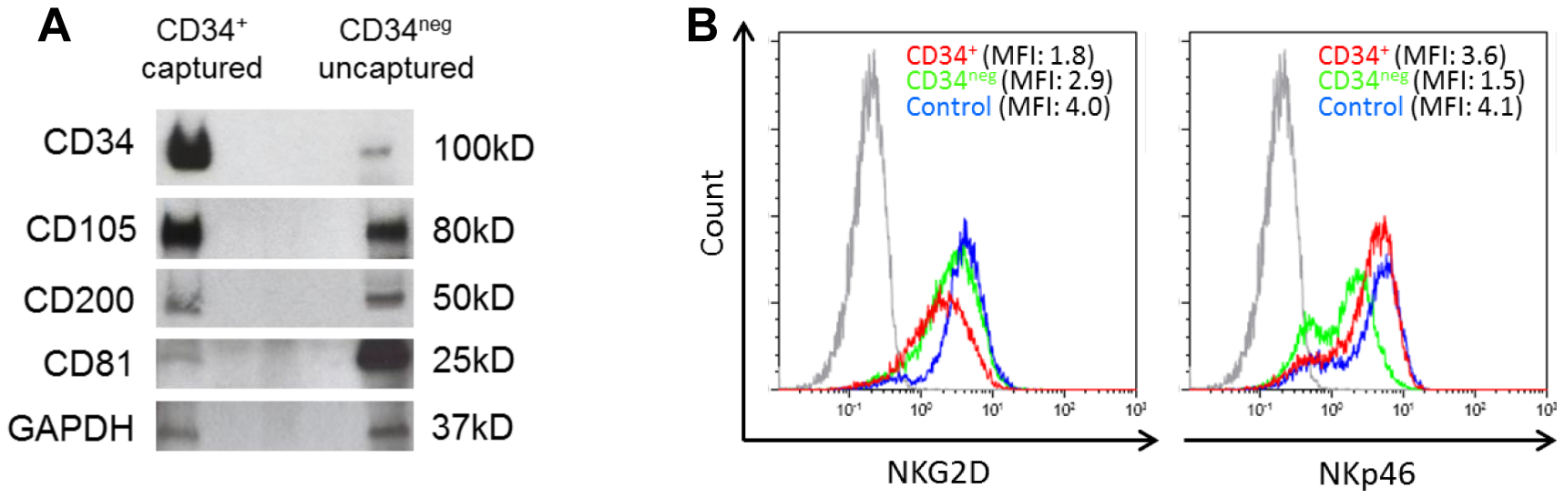
Characteristics of captured exosomes. A) A representative Western blot profile of the captured CD34^+^ exosomes and of non-captured CD34^neg^ exosomes which were isolated by ultracentrifugation from the same AML plasma sample. B) After co-incubation of NK cells with CD34^+^ exosomes captured directly from AML plasma or CD34^neg^ exosomes as described in materials and Methods, NKG2D expression (in MFI) was found to be down-regulated only with CD34+ exosomes (*red*) compared to CD34^neg^ exosomes (*green*) or control without exosomes (*blue*). The *gray* peak denotes isotype control. In contrast, NKp46 expression was down-regulated by co-incubation with CD34^neg^ exosomes.

Western blots of CD34^+^ exosomes captured from AML patients' plasma show that in addition to CD34, these exosomes carry proteins known as AML prognostic markers, including CD105 and CD200 (Figure 6A). The same markers were detected in exosomes captured from supernatants of the Kasumi-1 cell line (Figure 2D).

To determine whether the captured CD34^+^ exosomes are biologically active, purified normal human NK cells were co-incubated with captured CD34^+^ exosomes or CD34^neg^ exosomes and analyzed by flow cytometry for expression of NK cytotoxicity receptors. Captured CD34^+^ exosomes inhibited NKG2D expression in the NK cells, while CD34^neg^ exosomes reduced the expression of NKp46 (Figure 6B). These results indicate that the CD34^+^ and CD34^neg^ exosomes have differential effects on NK cells.

## Discussion

The rationale for development of the method for immunocapture of AML blast-derived exosomes from plasma of patients with CD34^+^ AML is based on existing evidence for selective ability of tumor-derived exosomes (TEX) to mediate immunosuppression [3]–[6]. Given that anti-tumor functions of immune cells are critical for the control of tumor progression in solid and hematological cancers [18], [19]; TEX-mediated immunosuppression could promote tumor growth and increase the rate of recurrence. Exosomes present in cancer patients' plasma are derived from normal as well as malignant cell, and true biologic effects of such mixed populations may be difficult to discern. As only TEX are expected to carry tumor-derived signals responsible for altering functions of immune cells, capture of TEX and their separation from the bulk of plasma exosomes is a necessary and potentially important step.

Several studies have previously tried to immunocapture exosomes from plasma. For example, the widely-advertised Exotest kit uses a sandwich ELISA in which anti-Rab-5b Abs are immobilized in wells of 96-well plates and the captured exosomes are detected with anti-CD63 Abs [20]. For capture of exosomes in supernatants of colon cancer cell lines, anti-A33 Ab- coated Dynabeads or anti-EpCAM Ab-coated microbeads have been used prior to proteomics-based analyses [21], [22]. These studies showed that the immunoaffinity-based capture was the most effective method for obtaining exosomes suitable for protein profiling, because these exosomes carried clinically-relevant tumor-specific proteins.

Based on this rationale and using somewhat different capturing strategy, we utilized AML patients' plasma, which was previously shown to be a rich source of exosomes [13], [14], to separate CD34^+^ from CD34^neg^ exosomes. Titration experiments with different quantities of the anti-CD34 Ab-coated beads and input exosomes allowed for quantification of the capture method and estimations of exosome recovery. In fact, the method allows for recovery of immunocaptured as well as non-captured exosomes and their comparisons in Western blots or functional assays. The identity of captured exosomes was confirmed by TEM. In addition, Western blots of captured exosomes showed they carried not only CD34 but also other AML blast markers in addition to the usual exosome markers. In AML, several markers are available for identification of leukemic blasts by flow cytometry or immunohistochemistry, including CD34, CD33, CD117, CD133, CD90, CD99, CCL-1 and others [2]. Among them, CD34 has been most extensively studied and shown to be associated with unfavorable outcome [23]. CD34^+^ blasts, like primitive leukemic stem cells, are resistant to apoptosis induced by chemotherapy and can transfer the disease in xenograft transplant models [2], [24], [25]. Although CD34 is expressed on normal hematopoietic precursor cells, AML blasts overexpress CD34, and only exosomes isolated from AML patients' plasma but not plasma of ND carried CD34. Therefore, selection of CD34 for immune capture of blast-derived exosomes is justified. However, only about 40% of AML patients have CD34^+^ blasts [26], [27]. For immunocapture of blast-derived exosomes in CD34^neg^ AML, beads coated with Abs to other blast markers, e.g., CCL-1 or CD99, or beads coated with a mix of several Abs could be used, pending a further modification of the capturing method. This strategy should allow for a broader application of this method to exosome capture in all AML patients.

We developed and optimized this procedure for immunocapture of CD34^+^ blast-derived exosomes from AML plasma in expectation that it will provide a specific tool for separation of tumor-derived immunologically-active exosomes from the bulk of those present in plasma and for discrimination of their potentially different biologic activities. Interestingly, preliminary experiments with NK cells used as targets for exosomes indicated that blast-derived captured exosomes had distinct biological activity from that mediated by non-captured exosomes. The former down-regulated NKG2D but not NKp46 expression levels, while the latter down-regulated NKp46 but not NKG2D expression levels. These data suggest that immunocaptured vs non-captured AML exosomes may have distinct biological effects. Having previously shown that TGF-β1carried by AML exosomes down-regulates NKG2D on NK cells [13], we are now attempting to show that these differential effects might reflect differences in TGF-β1 levels on CD34+ vs CD34negative exosomes. This is consistent with our hypothesis that isolation of blast-derived exosomes may be important for their use as future biomarkers. For AML patients with CD34^+^ blasts, this immunocapture method will now be used to perform molecular profiling of AML blast-derived exosomes and of non-captured, non-blast derived exosomes. This should indicate whether blast-derived exosomes could more effectively serve as indicators of the presence of leukemic blasts in the bone marrow or as biomarkers of response to therapies. We have already shown that the protein levels of total plasma exosomes and their molecular profiles dramatically change in the course of chemotherapy [14]. These data emphasized the potential clinical significance of AML plasma-derived exosomes (isolated but not selectively captured) as a sensitive biological measure of leukemic blast persistence after chemotherapy and as a potential predictor of relapse in AML. The ability to immunocapture blast-derived exosomes is expected to improve the diagnostic and prognostic potential of exosomes in AML.
